# Approaches to Account for Inter-Wave Mortality in Cognitive Aging Studies in High Mortality Settings

**DOI:** 10.1093/geroni/igaf122.1858

**Published:** 2025-12-31

**Authors:** Emma Nichols, Alden Gross, Erik Meijer, Jinkook Lee

**Affiliations:** University of Southern California, Los Angeles, California, United States; Johns Hopkins Bloomberg School of Public Health, Baltimore, Maryland, United States; University of Southern California, Los Angeles, California, United States; University of Southern California, Los Angeles, California, United States

## Abstract

A myriad of approaches exist to account for selective attrition from a study sample due to inter-wave mortality. When studies have longer inter-wave intervals or higher mortality, the importance of accounting for this potential bias increases. We compared mortality adjustment methods using data from Waves 1 (2017-2019) and 2 (2022-2024) of the Longitudinal Aging Study in India – Diagnostic Assessment of Dementia (LASI-DAD) (N = 3,546), given the high mortality in this setting, with 23.9% mortality during the 5-year inter-wave period. We estimated cognitive decline by age-group (60-69/70-79/80+) using a generalized estimating equations model and compared results to subsequent analyses applying inverse probability weights to account for death, using a joint model for decline and survival, and imputing cognitive status before death using informant data from end-of-life interviews. Given concerns of reporting biases in the exit interview data, we also considered adjustments to this data prior to imputation. Compared to the base model with no adjustment, estimated cognitive decline in the 80+ age group was 42.2%, 17.1%, 14.2%, and 42.1% greater for the model with IPW, joint model, model with imputations, and model with adjusted imputations, respectively. IPW-based estimates were more variable (i.e., had larger standard errors) than other methods. The percentage difference was smaller for age groups with lower mortality (e.g., 13.9% for the model with adjusted imputations in the 60-69 age group). Results highlight the potential impact of accounting for mortality. Comparisons of methods suggest some heterogeneity in estimates and highlights potential of using imputations with adjusted end-of-life interview data.

